# Endoscopic endonasal transsphenoidal surgery in children: widening the spectrum of oncological indications in the pediatric age group

**DOI:** 10.1007/s00381-025-06983-2

**Published:** 2025-10-15

**Authors:** V. Mastropasqua, M. Rigante, L. Massimi, P. Frassanito, F. Bianchi, M. Farina, G. Tamburrini

**Affiliations:** 1 Pediatric Neurosurgery, Fondazione Policlinico Universitario A. Gemelli IRCCS, Rome, Italy; 2 Otolaryngology and Head-Neck Surgery, Fondazione Policlinico Universitario A. Gemelli IRCCS, Rome, Italy; 3 Neurosurgery, Università Cattolica del Sacro Cuore, Rome, Italy; 4 Otolaryngology and Head-Neck Surgery, Università Cattolica del Sacro Cuore, Rome, Italy

**Keywords:** Children, Endoscopy, Surgery, Tumor

## Abstract

**Purpose:**

Endoscopic endonasal transsphenoidal surgery (EETS) is increasingly used in children to treat skull base lesions. The aim of this study is to present our updated institutional experience.

**Methods:**

A retrospective analysis of all pediatric patients (≤18 years old) treated via EETS for skull base pathologies at our institution, between January 2006 and December 2022, was carried out.

**Results:**

Sixty-seven children (mean age: 11.4 years) were operated on. Overall, 85 procedures were performed, including tumor residuals/recurrences after a previous craniotomy approach (11). Considering the planned goal of surgery, GTR was achieved in 73% of the cases, while STR and PR in 100% of the cases. Pituitary adenoma was the most common histotype (20), followed by craniopharyngioma (19), chordoma and sarcoma (8), optic pathway/hypothalamic glioma (5), Rathke cleft cyst (4), germ cell tumor (3), fibrous dysplasia (3), and angiofibroma (2). After surgery, most patients experienced improvement or stability of the preoperative symptoms. No surgical mortality or late nasal and facial complications were observed. Postoperative CSF leak occurred in 10 cases (11.7%). Postoperative CSF infection happened in 4 cases (6%). Six patients (12%) presented new-onset diabetes insipidus after surgery. A total of 21 patients (31.3%) had at least 1 reoperation for recurrent or progressive disease, consisting of EETS in 17 cases (68%). Eighteen patients (26.9%) with residual and/or malignant tumors received adjuvant chemotherapy and/or radiotherapy.

**Conclusions:**

EETS provides a safe and effective minimally invasive approach to treat a wide spectrum of skull base lesions in the pediatric age group.

## Background

Skull base tumors in the pediatric population are relatively rare, but their course and their surgical management can produce significant morbidity [1, 2]. In the past, they have been approached mainly through craniotomies or combined craniofacial resections, which may require brain retraction and damage the growing regions of a developing craniofacial skeleton, thus causing potential long-term sequelae [3–5]. More recently, endoscopic endonasal transsphenoidal surgery (EETS) has offered an important, less invasive surgical option, providing direct access to midline anterior skull base lesions—the most frequent in the pediatric population—with remarkable intraoperative visualization and magnification [6, 7]. Therefore, this approach has become increasingly used in the last two decades to treat a wider and wider spectrum of tumors, even in children [8, 9]. Despite some challenging aspects compared with adults (namely, narrow anatomical corridors, incompletely pneumatized sinuses, and interference with craniofacial development), a growing body of literature has described the safety, feasibility, and effectiveness of EETS [2, 10–16]. In this article, we report an update on the experience of our center compared with a decade ago, and we discuss outcomes and complications after an increased understanding and longer follow-up time [17].

## Methods

### Patient population

A retrospective analysis of all pediatric patients (≤18 years old) consecutively admitted and treated via endoscopic endonasal transsphenoidal surgery for skull base pathologies at our institution between January 2006 and December 2022 was carried out. During this period, 67 patients were identified. As outlined in the results, some of these patients underwent previous and/or subsequent procedures using other approaches (craniotomy or transventricular endoscopy).

### Study measures

Operative notes, discharge letters, and histological reports were collected to obtain information about patients’ demographics, clinical presentation, surgical details, histological diagnoses, and outcomes. The extent of resection was defined according to the intraoperative view and postoperative imaging, and on clinic-radiological follow-up. The patients’ data were analyzed using a statistical software (2025 JMP® JMP Statistical Discovery LLC, Cary, NC).

### Pre-surgical assessment

The clinical preoperative work-up consisted of physical examination, ophthalmologic and endocrinologic evaluation, and ENT assessment of the nasal cavities to estimate the feasibility of the endonasal approach. All the patients were investigated through hormonal screening, brain MRI, and a CT scan of the anterior skull base. This was utilized to point out possible anomalies of the anatomical bone landmarks. Neuroimaging for neuronavigation was obtained and used in selected cases (e.g., non-pneumatized sphenoid sinus).

### Surgical details

A team that included ENT surgeons and neurosurgeons carried out all the operations. The procedures were realized with the patient in a supine position and the head elevated (10–30°) and slightly tilted to the right. The “two-nostrils-four-hands” technique [18] was routinely used as well as the “diving technique” [19], which was applied to improve the visualization of the surgical field. The best surgical route to approach the sphenoid sinus (direct paraseptal, transethmoid-sphenoidal, or transethmoid–transpterygoid-sphenoidal) was chosen according to the tumor extension and the surgical anatomy. The unilateral removal of the middle turbinate was rarely required. In the case of the conchal variant of the sphenoid sinus, the sphenoid bone was drilled to reach the sella. This was realized using a high-speed drill and CT-guided neuronavigation. An extended approach to the clivus was performed for clival masses. At the end of the surgical procedure, a multi-layered reconstruction of the sellar floor with autogenic materials (septal or turbinate mucoperiosteum, septal cartilage, turbinate bone, abdominal fat) and/or commercially available dural substitutes and nasoseptal flap (NSF) was performed, as explained in the results.

### Post-surgical follow-up

All the patients underwent a strict clinical and radiological follow-up, including endoscopic evaluation 2–3 weeks after surgery, to verify the correct re-growth of the mucosal layer in the sphenoid sinus, the effectiveness of the sinus ventilation, and the presence of synechiae or crusts to be removed; neuro-radiological follow-up with brain MRI (3–6–12 months after surgery and then yearly); hormonal tests in the postoperative time and specific endocrinological follow-up; early (3 months) and yearly visual acuity and visual field evaluation in patients affected by visual impairment.

## Results

### Demographics

Overall, 85 EETS procedures were performed in 67 patients. The mean age at surgery was 11.4 years (range 3–18 years). The male/female ratio was 1. The mean postoperative hospital stay was 7 days. Eleven patients (16.4%) were previously operated on by a craniotomy approach and underwent EETS for recurrent or progressive disease. Three patients underwent planned staged surgical procedures: a 9-year-old patient affected by clival chordoma, who underwent transclival EETS as the first stage, suboccipital craniotomy as a second stage, then proton beam radiation; a 10-year-old patient also affected by clival chordoma, who underwent right retrosigmoid craniotomy as the first stage, transclival EETS as the second stage, then a redo retrosigmoid craniotomy for recurrence in that location; a 9-year-old patient affected by craniopharyngioma, who underwent endoscopic transventricular resection as the first stage and EETS as the second stage.

### Pathology

Pituitary adenoma was the most common histotype (29.9%), followed by craniopharyngiomas (28.4%), chordomas/sarcomas (11.9%), optic pathway/hypothalamic gliomas (7%), germ cell tumors (4.5%), nasopharyngeal angiofibromas (3%), and a not specified glioma with myxoid stroma. Besides, we treated Rathke’s cleft cysts (6%), fibrous dysplasia (4.5%), a pituitary stalk cyst, and Langerhans cell histiocytosis. Details on histological diagnosis are summarized in Table 1.

**Table 1:** **Pathology**

Pathology	*N* of patients
Pituitary adenoma	20 (29.9%)
Craniopharyngioma	19 (28.4%)
Chordoma/sarcoma	8 (11.9%)
Optic pathway/hypothalamic glioma (OPHG)	5 (7%)
Rathke cleft cyst (RCC)	4 (6%)
Fibrous dysplasia	3 (4.5%)
Germ cell tumor (GCT)	3 (4.5%)
Angiofibroma	2 (3%)
Other pathologies	3 (4.5%)

It is important to underline that a multidisciplinary tumor board, including oncologists, radiotherapists, and pediatricians, was convened preoperatively in all cases of optic pathway/hypothalamic gliomas, and the surgical indication was given for lesions larger than 3 cm, exerting mass effect on the optic pathways and/or causing symptoms of intracranial hypertension, as well as to find specific molecular markers (e.g., BRAF) for targeted therapies. As reported in Table 6, the surgical goal for OPHG, set after the multidisciplinary meeting and achieved in all cases, was always a subtotal or partial resection in order to decompress the optic pathways, get the histological diagnosis, and improve the efficacy of subsequent adjuvant therapies.

Rathke’s cleft cysts were operated on when they had the radiologic appearance of craniopharyngiomas. Pituitary adenomas were surgically treated in case of adenomas causing hypopituitarism and/or visual impairment, in case of Cushing disease, gigantism, sex hormone disorders, and/or drug resistance for macroprolactinomas. Germ cell tumors were operated on in the absence of coexisting pineal lesions and with negative markers in serum and CSF.

### Clinical presentation

The most common presenting symptoms were headache, visual impairment, endocrinological problems related to pituitary impairment (growth retardation, diabetes insipidus, obesity, amenorrhea, galactorrhea, Cushing disease), neurological impairment, rhinorrhea, and epilepsy. They are summarized in Table 2.

**Table 2:** **Clinical presentation**

Presenting symptom	Pituitary adenoma	Craniopharyngioma	RCC	Chordoma/sarcoma	OPHG	GCT
Headache	46%	42%	50%	38%	–	100%
Visual impairment	31%	47%	–	63%	80%	50%
CN deficit	–	5%	–	38%	–	–
Anterior pituitary disorders	100%	83%	75%	–	20%	50%
Diabetes insipidus	–	44%	50%	–	20%	50%
Nausea/vomiting	–	22%	25%	–	–	100%
Epilepsy/seizures	8%	5%	–	–	–	–
Paresis	–	–	–	25%	–	–

### CSF leak grading and reconstructive techniques

As previously described by Kelly[20], the intraoperative CSF leak was graded 1 in 11.5%, 2 in 71.4%, and 3 in 17.1% of the cases where an opening of the suprasellar cistern occurred. The grade 3 CSF leak rate was directly related to the high percentage of suprasellar lesions (43.5%) in the present series. The grading system guided the type of sellar floor reconstruction, which was always multi-layered, including an NSF in 50% of grade 1 cases, an NSF in 71.4% and fat in 9.5% of grade 2 cases, and an NSF in 100% and fat in 16.7% of grade 3 cases. The steps and materials used for skull base repair according to CSF leak grade are detailed in Table 3, as are the pathologies in each category. As expected, higher leak grades correspond to more aggressive and invasive pathologies, like craniopharyngiomas, chordomas, and sarcomas.

**Table 3:** **Reconstructive techniques**

Leak grade	Protocol of repair	% of patients*	Pathologies
1	1. Intrasellar spongostan 2. Bone buttress (nasal septum or turbinate fragment) onlay plus glue 3. Eventual NSF (if persistent leak) plus glue 4. Spongostan and binostril Merocel swabs	11.5%	Pituitary adenoma (75%) RCC (25%)
2	1. Eventual intrasellar fat (if large dural defect) 2. Intrasellar spongostan 3. Dural substitute inlay plus glue 4. Bone buttress (nasal septum or turbinate fragment) onlay plus glue 5. Eventual NSF (if persistent leak) plus glue 6. Spongostan and binostril Merocel swabs	71.4%	Pituitary adenoma (8%) OPHG (28%) Craniopharyngioma (60%) GCT (4%)
3	1. Eventual intrasellar fat (if large dural defect) 2. Intrasellar spongostan 3. Dural substitute inlay plus glue 4. Bone buttress (nasal septum or turbinate fragment) onlay plus glue 5. NSF plus glue 6. Spongostan and binostril Merocel swabs	17.1%	Pituitary adenoma (17%) Chordoma/sarcoma (67%) GCT (16%)

^*^Where an opening of the suprasellar cistern occurred

Intraoperative lumbar drainage was placed, combined with the multi-layered reconstruction, in 14.3% of the cases, all with grade 3 CSF leakage, after tumor removal and with clinical and/or anatomical risk factors (lower age, insufficient tissues, recurrent tumors) for postoperative CSF leakage. Lumbar drains were typically placed immediately after the surgical procedure and were removed before discharge in the absence of CSF leakage. Intraoperative lumbar drains were mainly used at the beginning of our experience to secure particularly difficult sellar floor reconstructions during the initial phase of the learning curve in EETS. As underlined in the following sections, since 2015, we have stopped placing them intraoperatively in consideration of the excellent results provided by the multi-layered repair alone.

### Complications

Table 4 shows the main complications reported in the peri- and postoperative period, divided according to pathology. Craniopharyngioma was associated with the highest rate of postoperative complications. The most common event was CSF leakage (10 cases out of 85, 11.7%), managed with transient external lumbar drainage in all cases (in four of them, an endoscopic endonasal revision of the sellar repair was performed).

**Table 4:** **Outcomes and complications**

Outcomes and complications	Pituitary adenoma	Craniopharyngioma	Chordoma/sarcoma	OPHG	GCT	RCC	Angiofibroma	Fibrous dysplasia	Others	Tot
Visual outcome										
Improved*	1 (25%)	3 (33.3%)	3 (100%)	1 (25%)	1 (100%)	–	–	–	1 (50%)	10 (43.5%)
Stable	14 (93.3%)	13 (76.5%)	5 (71.4%)	4 (80%)	1 (50%)	4 (100%)	3 (100%)	3 (100%)	2 (66.7%)	49 (83.1%)
Worsened	0/15	1 (5.9%)	0/7	0/5	0/1	–	–	–	–	1 (1.7%)
AP function			–							
Improved**	10 (71.4%)	1 (7.1%)	0/7	0/5	0/1	1 (33.3%)	0/3	0/3	0/3	12 (37.5%)
Stable	5 (33.3%)	13 (76.5%)	7 (100%)	5 (100%)	2 (100%)	3 (75%)	3 (100%)	3 (100%)	3 (100%)	44 (74.6%)
Worsened	0/15	3 (17.6%)	0/7	0/5	0/1	–	0/3	0/3	0/3	3 (5.1%)
PP function			–							
Stable	14 (93.3%)	13 (76.5%)	7 (100%)	5 (100%)	1 (50%)	4 (100%)	3 (100%)	3 (100%)	3 (100%)	53 (89.8%)
DI improved	–	–	–	–	–	–	–	–	–	–
DI worsened	–	–	–	–	–	–	–	–	–	–
DI new onset***	1 (6.7%)	4 (36.4%)	0/7	0/5	1 (100%)	0/2	0/3	0/3	0/3	6 (12%)
CSF leakage post-op	1 (5.9%)	6 (24%)	0/7	1 (16.7%)	1 (50%)	1 (25%)	0/3	0/3	0/3	10 (11.7%)
Meningitis/CSF infection	–	1 (4%)	1 (14.3%)	1 (16.7%)	–	1 (25%)	0/3	0/3	0/3	4 (6%)
Neurological deficit										
Improved	–	0/1	3 (60%)	–	1 (100%)					4 (57.1%)
Stable	–	1 (100%)	1 (20%)	–	–					1 (33.3%)
Worsened	–	0/1	1 (20%)	–	–					1 (16.7%)
Hydrocephalus	–	–	2 (28.6%)	–	–					2 (2.4%)
Vasospasm	–	–	1 (14.3%)	–	–					1 (1.2%)
Hemorrhage/ischemia	–	–	1 (14.3%)	–	–					1 (1.2%)

^*^Percentage based on patients with only preoperative visual disturbance

^**^Percentage based on patients with only preoperative anterior pituitary disfunction

^***^Percentage based on patients without preoperative diabetes insipidus

CSF infection occurred in four patients and was controlled with antibiotic therapy. Six patients presented new-onset diabetes insipidus, while salt-wasting syndrome occurred in one patient. Two patients developed acute hydrocephalus in the postoperative period: one case (5-year-old female treated for a clival chordoma) occurred after intraoperative bleeding and required external ventricular drainage, then bifrontal craniectomy to treat intracranial hypertension, and finally a ventricular-peritoneal shunt after the cranioplasty; the other case (4-year-old male treated for an epithelioid sarcoma), possibly following a mild subarachnoid hemorrhage, required only temporary external ventricular drainage. Finally, one patient showed a transient worsening of a recurrent and remitting sixth cranial nerve palsy, which recovered after systemic corticosteroid therapy.

Comparing the 2006–2015 and 2016–2022 periods and considering the major complications (i.e., postoperative CSF leakage, CSF infection, and new-onset diabetes insipidus), we can see a remarkable reduction in 2 out of 3 items, as detailed in Table 5. This is related to a more extensive use of neuronavigation systems, the development of more precise endoscopic instruments, and the improvement and standardization of the multi-layered reconstruction, as detailed in Table 3.

**Table 5:** **Comparison between 2006–2015 and 2016–2022 periods**

Major complications	2006–2015 period	2016–2022 period
Post-op CSF leakage	6/38 (15.8%)	2/30 (6.7%)
CSF infection	2/38 (5.3%)	2/30 (6.7%)
DI new onset	3/38 (7.9%)	1/30 (3.3%)

### Outcomes

After surgery, most patients experienced an improvement or stability of the preoperative symptoms, as reported in Table 4. Intracranial hypertension syndrome promptly recovered after the operation in all patients; no worsening of preoperative neurological deficit nor new alterations were noticed postoperatively, apart from the first case of acute hydrocephalus, who developed a severe neurological worsening with a persistent minimally conscious state. Visual impairment improved in ten cases, worsened in one case for disease progression during long-term follow-up, and remained stable in the others.

Cranial nerve palsy disappeared in three cases [21] and improved in the remaining one; epileptic seizures regressed, apart from the aforementioned complicated case. Preoperative anterior hypopituitarism improved in 12 patients, worsened in three, and remained stable in the others. Hormone hypersecretion regressed in all cases except one that considerably improved. Overall, 46 patients (69.7%) currently rely on chronic hormone replacement. The surgical mortality was nil.

**Fig. 1:**
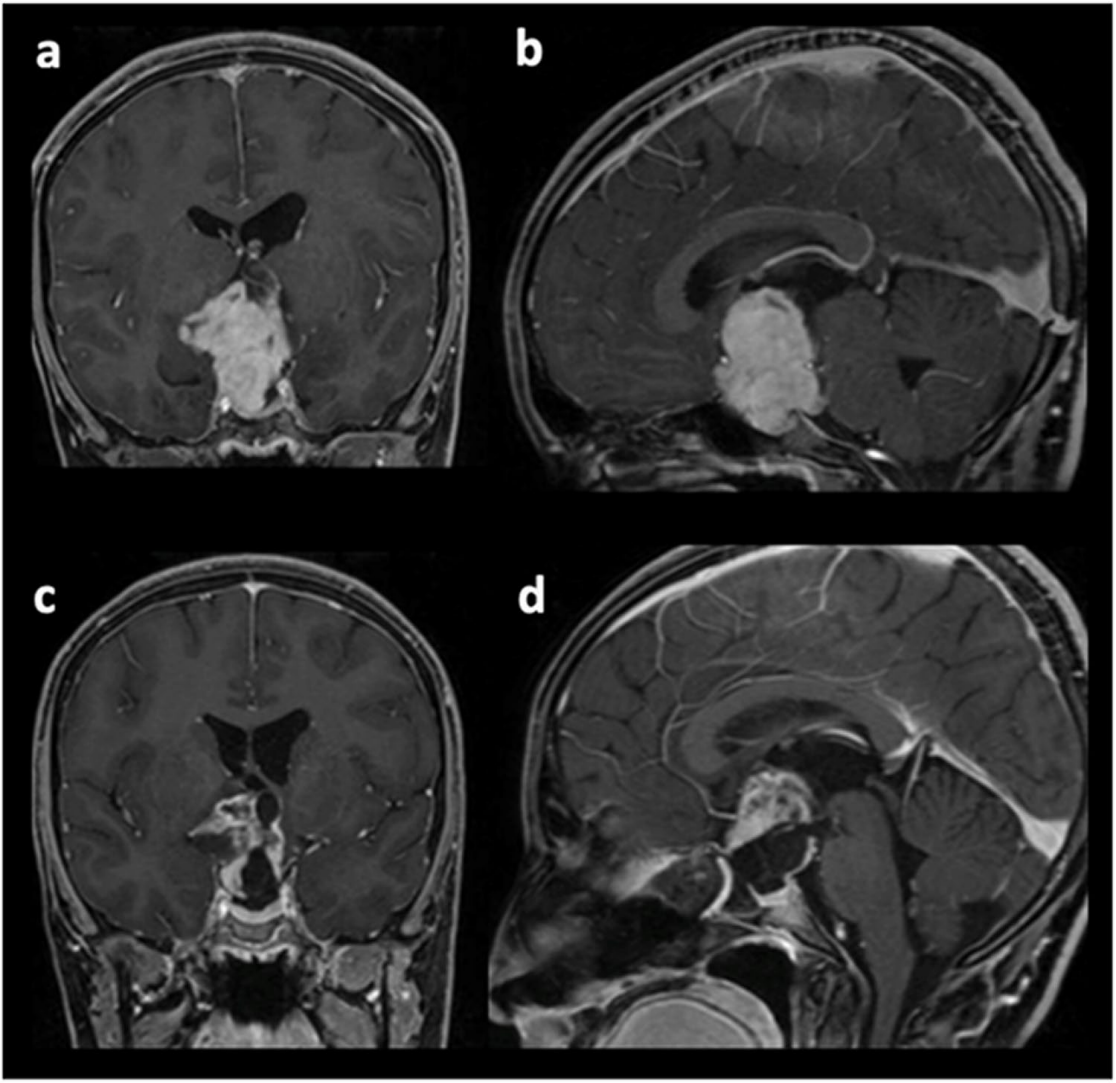
Pilocytic astrocytoma of the optic pathways in a 15-year-old female: **a, b** coronal and sagittal preoperative T1-weighted contrast-enhanced MRI; **c, d** coronal and sagittal postoperative imaging, showing planned partial resection and optic pathways decompression

**Fig. 2:**
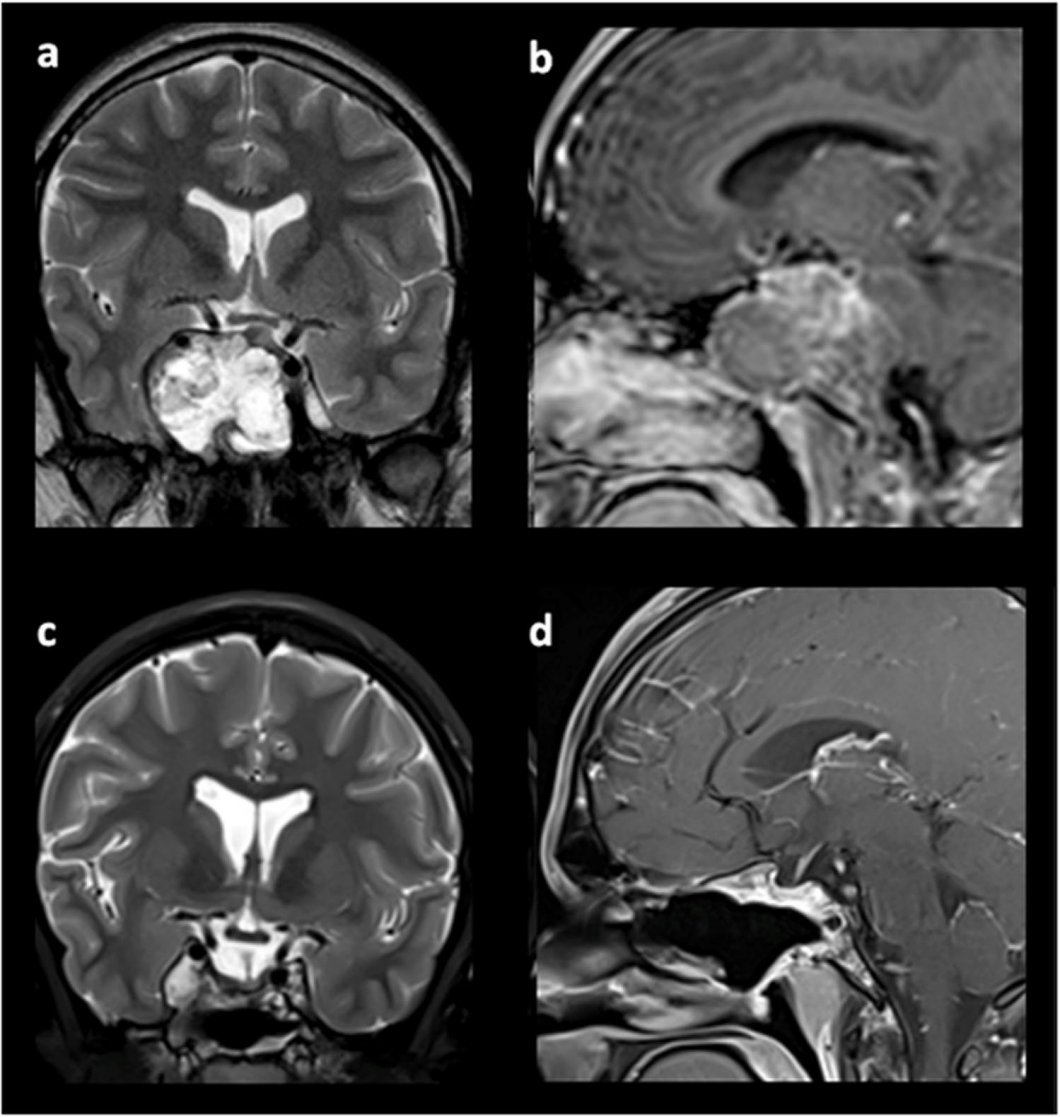
Sphenopetroclival chondrosarcoma in a 16-year-old male: **a**, **b** coronal T2-weighted and sagittal T1-weighted contrast-enhanced MRI preoperative imaging; **c**, **d** coronal and sagittal postoperative imaging, showing gross total resection

**Fig. 3:**
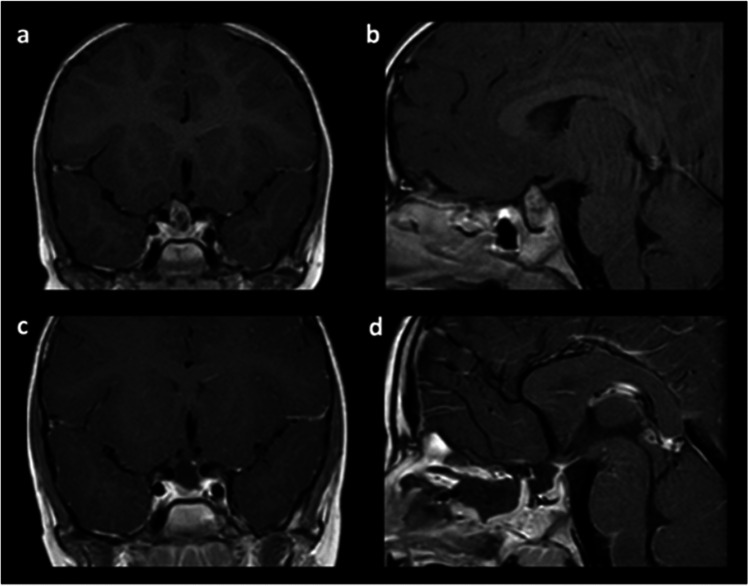
Sellar and suprasellar adamantinomatous craniopharyngioma in a 4-year-old female: **a**, **b** coronal and sagittal T1-weighted contrast-enhanced preoperative MRI; **c**, **d** coronal and sagittal postoperative MRI, showing gross total resection

Eighty-three (97.6%) EETS procedures were performed for tumor resection, while two procedures were performed for postoperative CSF leak. Considering the planned goal of surgery, GTR was achieved in 73% of the cases, while STR and PR were achieved in 100% of the cases. Overall, a gross total resection of the tumor was achieved in 46 cases (55.4%). In comparison, a subtotal and a partial removal were realized in the remaining 21 (25.3%) and 16 cases (19.3%), respectively, as detailed in Table 6. Some examples of the surgical outcomes in different pathologies are provided in Figs. 1, 2, and 3.

**Table 6:** **Extent of resection and adjuvant therapy**

Pathology	EOR (achieved/attempted, achieved/total)			Adjuvant therapy		Reoperation
	Gross total	Subtotal	Biopsy/decompression	Radiotherapy	Chemotherapy	
Pituitary adenoma	17/22 (77%), 17/23 (73.9%)	−, 5/23 (21.7%)	1/1 (100%) 1/23 (4.3%)	2 (10%)	1 (5%)	2 (10%)
Craniopharyngioma	14/24 (58.3%), 14/27 (51.9%)	−, 9/27 (33.3%)	3/3 (100%), 4/27 14.8%)	4 (21.1%)	–	10 (52.6%)
Chordoma/sarcoma	5/6 (83.3%), 5/11 (45.5%)	1/1 (100%), 2/11 (18.2%)	3/4 (75%), 3/11 (36.4%)	6 (75%)	1 (12.5%)	3 (37.5%)
OPHG	−, 0/6	2/2 (100%), 2/6 (33.3%)	4/4 (100%), 4/6 (66.7%)	–	3 (60%)	2 (40%)
GCT	−, 0/3	2/2 (100%), 2/3 (66.7%)	1/1 (100%), 1/3 (33.3%)	1 (33.3%)	2 (66.7%)	2 (66.7%)
RCC	3/4 (75%), 3/4 (75%)	−, 1/4 (25%)	–	–	–	1 (25%)
Angiofibroma	3/3 (100%), 3/3 100%)	–	–	–	–	1 (50%)
Fibrous dysplasia	3/3 (100%), 3/3 100%)	–	–	–	–	–
Other pathologies	1/1 (100%), 1/3 (33.3%)	–	2/2 (100%), 2/3 (66.7%)	–	1 (33.3%)	–
Total	46/63 (73%), 46/83 (55.4%)	4/4 (100%) 21/83 (25.3%)	14/14 (100%) 16/83 (19.3%)	13 (19.4%)	8 (11.9%)	21 (31.3%)

### Follow-up

The duration of the postoperative clinical follow-up ranged from 6 to 182 months (median 70 months). Eleven patients received short-term follow-up (i.e., 3 months) due to relocation to other cities and/or referral to local physicians.

The overall mortality was 1.5% (1 patient) and was related to disease progression: a 4-year-old patient affected by epithelioid sarcoma underwent two EETS followed by chemotherapy and radiotherapy and died 1 year later.

A total of 21 patients (31.3%) had at least one reoperation for recurrent or progressive disease. Planned second-stage procedures (either open or endoscopic) were excluded from this analysis. Of the 21 patients undergoing reoperation, 18 (85.7%) had one additional surgery, whereas 3 (14.3%) required two or more reoperations.

The reoperation surgical approach consisted of EETS in 17 cases (68%) and open craniotomy in 8 cases (32%). The most common pathologic diagnosis requiring reoperation was craniopharyngioma (52.6%), followed by chordomas/sarcomas (37.5%). Eighteen patients (26.9%) with residual and/or malignant tumors received adjuvant therapies: 10 patients received radiotherapy, five patients received chemotherapy, and three patients were treated with both chemotherapy and radiotherapy.

Most of the patients recovered well after surgery and, during the follow-up period, presented Karnofsky/Lansky scores >90% in more than 95% of cases. None of the patients presented abnormal craniofacial growth at periodic physical examinations.

## Discussion

In the last two decades, EETS has been increasingly used to treat pediatric skull base lesions, with safety, feasibility, and efficacy comparable to those of the adult population, as reported in the literature [2, 10–13, 16, 22]. Surgical instrumentation development, technical skills improvement, and increasing experience among neurosurgeons and otolaryngologists at tertiary pediatric centers have allowed this vital progress.

Our results confirmed the high versatility of this approach, showing its applicability in a broad spectrum of skull base lesions, as reported in Table 3. EETS provided safe corridors to reach sellar, parasellar, and suprasellar pathologies, extending to the third ventricle, the petrous apex, and the retroclival space.

According to the different pathologies, this surgery proved effective as a stand-alone approach as well as a part of staged procedures in combination with craniotomies, providing satisfactory outcomes in both cases. These results, already predictable in our previous series, are consistent with the other ones then published in literature: Chivukula et al. [2], authors of the most extensive available series, as well as Yang et al. [10], Kahilogullari et al. [13], and Quon et al. [12] showed the high number and variability of tumors, which can be approached via EETS.

It is difficult to compare the craniotomy approaches to EETS because the indications are different. In our experience, we performed the pterional approach for lesions with a lateral extension in the middle fossa and the retrosigmoid approach for lesions located laterally in the posterior fossa: in these cases, the EETS as a stand-alone approach could not allow a complete visualization and control of the regional anatomy, preventing a safe and effective surgery. We never used the interhemispheric transcallosal transventricular approach, because the main component of the listed pathologies was centered on the skull base and, even when they extended into the third ventricle (i.e., in the case of craniopharyngiomas), the EETS allowed us to reach it and effectively manage the intraventricular component. Besides, the risks of the interhemispheric transcallosal transventricular approach are well known [23, 24]: damage to the bridging cortical veins, to the thalamostriate veins, and/or to the internal cerebral veins with possible consequent drowsiness, hemiplegia, and mutism, especially if opening the choroidal fissure to access the middle and posterior third ventricle; damage to the fornices with possible memory deficit; increased risk of seizures; rarely disconnection syndrome in the case of extended callosotomy.

In this case series, EETS allowed us to achieve GTR in 73%, while STR and PR in 100% of the cases in which these were set as primary goals; the rates of GTR and STR across all pathologies were 55.4% and 25.3%, respectively. A total of 21 patients (31.8%) had at least one reoperation for recurrent or progressive disease. Compared to our previous series, which showed better GTR rates, these data were affected by the number of highly invasive pathologies treated, such as craniopharyngiomas, chordomas/sarcomas, and germ cell tumors, for which subtotal or partial resection was planned as a primary goal in most cases. Considering pituitary adenomas, Rathke cleft cysts, fibrous dysplasia, and angiofibroma, we obtained significantly higher GTR rates.

Our results in terms of extent of resection are relatively similar to the ones from Chivukula et al. [2] but inferior to the ones from Yang et al. [10], authors of the second largest available series. In particular, the latter authors had better GTR rates for craniopharyngiomas, but higher rates of aggravated anterior pituitary function and new-onset diabetes insipidus. Additionally, the majority of craniopharyngiomas we could not completely resect presented small calcifications strictly adherent to major vessels, which we preferred to leave behind to avoid vascular complications. Another essential difference between the other series and ours is that we treated a relevant number of lesions consistent with optic pathway/hypothalamic gliomas to obtain histological diagnosis and optic pathway decompression through partial resection. This is another reason for our relatively low GTR rate across all pathologies.

Considering primary EETS surgeries versus redo EETS surgeries, GTR rates decrease in the latter, as outlined in Table 7. This is consistent with the results reported in the literature [11].

**Table 7:** **Comparison between primary and redo surgeries**

	Primary EETS (62)	Redo EETS (16)
Gross total	37 (59.7%)	9 (56.3%)
Subtotal	14 (22.6%)	6 (37.5%)
Partial	11 (17.7%)	1 (6.25%)

Regarding neurological and endocrine outcomes, as illustrated in Table 4, most patients presented improvement or no change in their pituitary function, vision, and neurological performance.

These data confirm the findings reported in our previous paper and in the literature [2, 10, 13]. Overall, new-onset diabetes insipidus was 15.8%, similar to the results from Chivukula et al., but inferior to the findings from Yang et al., probably because the latter was more aggressive, especially in the case of craniopharyngiomas (86% vs 36% of new-onset DI).

Our study strengthened the safety of EETS, broadly showing low complication rates, as reported in Table 4. The most frequent complication was postoperative CSF leakage, occurring in 11.9% of cases. This rate is similar to what was reported by Chivukula et al. (12.5%) and Behbahani et al. (12.9%). Kahilogullari et al. reported a rate of 1.8%, but their series included fewer invasive tumors compared to ours; Yang et al. obtained probably the best rate (2%), using, in some cases, buttress-type autologous fascia plus nasoseptal flaps with the need for an additional skin incision, and in others, multiple onlay artificial materials with some cases of aseptic meningitis. Although there is still debate about the best reconstruction protocol, it is generally accepted the paramount role of the pedicled nasoseptal flaps in postoperative CSF leakage prevention, especially in the case of large defects, high flow intraoperative CSF leaks, and revision cases [25–27], also in very young children [28], as confirmed in our experience. Besides, this important breakthrough has reduced the need for lumbar drains in the perioperative period [29–31], as we have experienced in our recent practice.

Similar to other reported series, infectious complications occurred in 5% of cases [2, 11, 12]. Yang et al. [10] reported a higher rate of meningitis (12%), but, as previously mentioned, most were aseptic.

The kind and rate of complications change according to pathology because they are related to specific features, such as location, vascularity, and biological behavior: among the most represented pathologies, craniopharyngiomas were associated with the highest rate of complications, whereas pituitary adenomas with the lowest one, in particular considering new-onset diabetes insipidus and postoperative CSF leak (Tables 4 and 5). This is consistent with the other main series previously mentioned [2, 10].

In general, EETS was very well tolerated by our patients compared to traditional approaches, thanks to decreased surgical time and length of stay, reduced postoperative pain, and minimal or absent visible scars. Prompt recovery also allowed fast starting of adjuvant therapies when required. These findings confirm the reports from other series in the literature [11, 13] and our previous reports [17]. For these reasons, the present study sustains the results from Stapleton et al. [32]: EETS is a cost-effective model for the removal of skull base lesions in the pediatric population.

Some studies in the literature expressed concerns about anatomical limitations in the pediatric population, namely disruption of craniofacial growth centers, small piriform aperture width, reduced intercarotid distance, and absent or incomplete sinus pneumatization [33, 34]. Regarding the first one, we found no craniofacial growth anomalies during our follow-up period, according to other papers in the literature [2, 11, 35]. Smaller nostrils and narrower sino-nasal corridors have been properly overcome with dedicated surgical instrumentation, confirming the feasibility of the approach in very young children [36]. The early maturation of intercarotid distance precludes this from being an anatomic limitation [31]. About the last concern, it is demonstrated that sphenoid pneumatization begins as early as the first months of age and usually ends within the first decade [37], following an inferior-to-superior and lateral direction and significantly influencing parameters like the intercarotid distance, the transsphenoidal working angle, and the drilling distance to reach the sellar floor [38]. In our experience, we found three patients with incomplete pneumatization of the sphenoid sinus, and we could successfully treat them thanks to detailed preoperative radiological assessment and accurate, navigation-guided drilling to access sellar and parasellar lesions. Kuan et al. [39] reported that the lack of sphenoid pneumatization does not affect EETS outcomes in the pediatric population. It is thus not a contraindication for the EETS in pediatric patients. Nonetheless, skull base lesions can significantly impact normal skull base development and age-dependent growth patterns [38]. For this reason, it is paramount to carefully review the preoperative imaging and then choose the best approach.

### Limitations

This study presents the limitations of a retrospective review due to the lack of controlled prospectively collected data. Another key point is the heterogeneity of the pathologies treated, and the relatively small sample of patients considered for each one, which does not allow for drawing significant and definite conclusions about single pathological entities. Nonetheless, as stated by Chivukula et al. [2], understanding the efficacy and limitations of EETS in a population is impossible without considering all pathologies.

## Conclusion

EETS provides a safe and effective minimally invasive approach to treat a broad spectrum of skull base lesions in children. Controlled prospective multicentric studies with larger samples are needed to improve the knowledge and mastery of EETS among the different pediatric pathologies.

## Data Availability

Source data are available from the corresponding author upon reasonable request.
